# Mental health in women experiencing preterm birth

**DOI:** 10.1186/1471-2393-14-263

**Published:** 2014-08-09

**Authors:** Aud R Misund, Per Nerdrum, Trond H Diseth

**Affiliations:** Faculty of Health Sciences, University College of Oslo and Akershus, PO Box. 4, St. Olavs plass, N-0130 Oslo, Norway; Department of Clinical Neurosciences for Children, Women and Children’s Division, Oslo University Hospital, Rikshospitalet, PO Box 4950, Nydalen NO-0424 Oslo, Norway; Department of Medicine, Institute of Clinical Medicine, University of Oslo, PO Box 1171, Blindern 0318 Oslo, Norway

**Keywords:** Anxiety, Depression, Preterm birth, Psychological distress, PTSR

## Abstract

**Background:**

The aim of the study was to explore the degree of psychological distress, anxiety, and trauma related stress reactions in mothers who experience preterm birth. Secondarily, we wanted to identify possible predictors of maternal mental health problems.

**Methods:**

Twenty-nine mothers of 35 premature children born before 33rd week of pregnancy were assessed within two weeks after given birth. The standardized psychometric methods; Impact of Event Scale (IES), General Health Questionnaire (GHQ) and State Anxiety Inventory (STAI) assessed mental health problems. The predictors for maternal distress, anxiety, and trauma related stress reactions were pregnancy variables, preterm delivery, Gestation Age, maternal trait anxiety and parity. In addition, maternal prevalence of mental health problems was assessed by clinical diagnoses.

**Results:**

Our study revealed a high prevalence (52%) of posttraumatic stress responses in the mothers.

**Conclusions:**

Our results suggest an early examination of mothers’ psychological reactions to preterm birth at the maternity ward. An early intervention should be considered while the child still is in the neonatal intensive care unit.

## Background

In general, childbirth may become a traumatic event when intense fear, helplessness, pain and loss of control are experienced in labor and delivery [1, 2]. Many of these factors are likely to be present in a preterm birth situation. For mothers, the experience of giving preterm birth, and the subsequent experiences in the neonatal intensive care unit (NICU), may therefore cause substantial psychological distress. Exploring maternal mental health reactions following preterm birth is interesting from an attachment perspective as maternal mental health is known to affect children’s physical and mental development [3, 4]. Previous research has shown that women with preterm deliveries experience significantly higher levels of stress and depression than women who deliver at term [5–10]. However, less is known of maternal posttrauma reactions to preterm birth [11]. Stress is a psychological phenomenon that may present as anxiety, depression and trauma reactions. The co-morbidity of anxiety and depression in posttraumatic reactions is well known, but the knowledge of traumarelated stress reactions after preterm childbirth is still limited [12].

Behavioral and cognitive problems in prematurely born infants have been the main focus in research for several decades [13]. In addition to the severity of the child’s medical risk factors, parental mental health is known to contribute substantially to children’s cognitive, emotional, social and physical development [13–16]. A number of studies have explored maternal depression following preterm birth. This research has reported that preterm mothers are at higher risk of depression than term mothers shortly after preterm birth and that mothers with very-low-birth-weight infants have a continued risk for depression in the first postpartum year [10]. Furthermore, follow-up studies of term babies have shown that persisting parental depressive symptoms are an important predictor of child dysfunction [17, 18]. The predictors of postpartum depression that have achieved the greatest amount of consensus are previous psychiatric disorder, higher family disposal of psychiatric disorder, as well as poor social support, marital problems, and a higher amount of stress during pregnancy [1].

We know that about 1.5 to 3% of full term birth women show signs of posttraumatic stress disorder (PTSD) 6 months after giving birth, however, the maternal trauma reactions following a preterm birth is less explored. But the trauma and post trauma connection, however, is still unexplained [19].

One study revealed that a preterm birth experience may cause a long lasting traumatizing effect on parents; 49% of the mothers reported significant trauma reactions one year after delivery [20]. Muller-Nix et al. found a correlation between traumatic stress reactions and disturbances in mother-child interaction [21]. The strength of parents’ post-traumatic stress symptoms (PTSR) after preterm delivery is reported to be the most important predictor of the child’s sleeping and eating problems [22]. However, studies of mental health problems after preterm delivery are in general small non-randomized studies with differing trauma measurements.

The primary aim of the present study was to explore the degree of psychological distress, anxiety, and traumarelated stress reactions in mothers who deliver preterm. Secondarily, we wanted to explore the nature of maternal psychological distress and identify the predictors of maternal mental health problems.

## Methods

### Study design

This study used an explorative cohort design.

From June 2005 to July 2008 through two periods of 8 and 10 months, the psychological responses of 29 consecutive mothers of 35 premature children born before 33rd week of pregnancy at the Oslo University Hospital, Norway were assessed. The data collection was performed as soon as the mothers were able to attend the interview after preterm childbirth, (median 11 days (4–30)). Mothers of severely ill babies with uncertain survival and non-Norwegian speakers were not included. Medical charts and questionnaires were used to collect data about the childbirth, the child’s physical state, maternal previous mental health history, and socio-demographics.

The study group was a homogeneous group with high scores on socio-demographic variables like education, income, and housing standard. They all lived in a relatively affluent city district in the Oslo area. Most of them were giving birth first time late in their twenties or early thirties. All of them lived with the child’s father and none reported any relationship problems. Only two mothers were diagnosed with a chronic somatic illness. Twenty-eight percent of the mothers had become pregnant by IVF.

The babies in the study group had relatively high Apgar mean scores and only 23% of the babies needed mechanical ventilation for more than 24 hours.

The neonatal intensive care unit had applied several aspects of the newborn individualized developmental care and assessment program (NIDCAP) in their care for the preterm babies and in their parental support and supervising [23]. The parents included in the study were offered psychological care during hospital stay. Mothers with high levels of mental health reactions were referred to further psychological treatment after hospitalization.

### Measurements of maternal mental health problems

Maternal mental health problems were measured by the standardized psychometric methods Impact of Event Scale (IES), General Health Questionnaire (GHQ), and State/Trait Anxiety Inventory (STAI-X1/X2).

The 15-item version of the Impact of Event Scale (IES) [24] was used to assess behavioral aspects of distress. Clinically important stress related cognition and behavior were defined as an IES score ≥19. In this study, the stress factor was defined as *“Preterm childbirth”*. The IES-15 has two subscales measuring symptoms of intrusive psychological distress (7 items) and avoidant cognition and behavior (8 items). Intrusion is characterized by unbidden thoughts and images, troubled dreams, strong pangs or waves of feelings, and repetitive behavior. Avoidance responses include ideational constriction, denial of the meaning and consequences of the event, blunted sensation, behavioral inhibition or counter-phobic activity, and awareness of emotional numbness. The scoring range for each item is 0 (not at all) to 5 (very much). A subscale score of 0–8 usually denotes minor responses, 9–19 moderate responses and scores ≥20 denote severe responses. IES has been thoroughly validated and is one of the key psychometric assessments methods in traumatic stress research [24, 25].

The General Health Questionnaire (GHQ) [26] is a widely used screening instrument for assessing the presence of distress, psychopathology, and overall well-being, showing well established reliability and validity. The GHQ includes both positive and negative questions, and the short version GHQ-30 contains 30 items covering symptoms considered to reflect psychological distress and the subscales for depression and feelings of incompetence/low self-esteem (also referred to as well-being). Each question is answered on a four point scale. The answers to each item may be treated both as Likert sum score (recommended for use in longitudinal studies when measuring change) with weights assigned to each response (0-1-2-3) with a possible scale of 0–90, and as case sum score with weights (0-0-1-1) and possible range 0–30. When using the GHQ-30 as a screening instrument for overall psychological distress, as in this study, case-score has exhibited acceptable values for sensitivity and specificity. Clinically important psychological distress was defined as case total scores ≥6 [26–28].

The Spielberger State Trait Anxiety Inventory (STAI-X1 and STAI-X2) [29] was used to assess maternal anxiety***.*** STAI-X1 is a measure of state anxiety levels reflecting subjective feelings of tension, apprehension, nervousness and worry. STAI-X1 has a 20 item and a 12 item version. The 12 item STAI-X1 was used in the first data collection period and the 20 item STAI-X1was used in the second data collection period. Both versions consist of items eliciting to what extent the respondent is currently experiencing the symptom or sign: “Not at all”, “Somewhat”, “Moderately” or “Very much” and rated on a four-step scale (1-2-3-4) with a possible score range of 20–80 for the 20 item version and 12–48 for the 12 item version. Higher scores indicate more anxiety.

Ten items from the 20 items version are overlapping in the two versions (item no: 1, 2, 3, 5, 7, 11, 12, 13, 14, 15). A common 10 item STAI version was constructed for our analyses. For the 10 item version clinically important state anxiety was defined as a STAI score ≥20 (corresponding to ≥ 40 for 20 item version). STAI-X2 is a measure of trait anxiety that refers to individual differences in anxiety proneness i.e. in the tendency to see the world as dangerous and threatening, and in the frequency with which anxiety states are experienced. It consists of 20 items and the scoring range is 20–60. Clinically important significant trait anxiety was defined as ≥ 40.

STAI-X1 and X2 are reliable and widely used self-evaluation questionnaires that have been used in several studies in similar populations [29].

To explore the prevalence of anxiety, depression, and PTSR in particular, a tentative clinical diagnosis or not, based on the clinical diagnostic guidelines in the ICD-10 Classification of mental and behavioural disorders [30], was assessed by a psychiatrist (last author). The assessment was based on all information available in a clinical perusal of the psychometric self-reports IES, GHQ and STAI of each of the 29 preterm mothers, and blinded to the physical and socio-demographic characteristics of the mothers and their children.

### Statistical methods

Values of continuous variables are presented as means (SD) or if skewed as median and range. Categorical variables are given as proportions and percentages. Correlations between normally distributed and continuous variables were measured using Pearson’s correlation coefficient or Spearman’s correlation coefficient when the variables had a skewed distribution. Forward linear regression analysis was used to identify possible predictors of mental health and psychological distress within the study group. The three variables with the strongest bivariate associations were included in the multiple linear regression model. A careful check of the model assumptions, including an investigation of residual plots, did not reveal any violation of the assumptions. All analyses were performed in SPSS version 18. Two-sided statistical tests were applied, a 5% statistical significance level was chosen.

### Ethics

Written informed consent was obtained from participants prior to study start. The study protocol was approved by the Norwegian National Committee for Research Ethics (S-05068 and S-07096b) and by the Data Inspectorate (12360 and 07/1088). The study protocol was carried out in accordance with the Declaration of Helsinki.

## Results

Twenty-nine of 34 families (85.3%) that met the inclusion criteria were included in the study group. Five mothers refused to participate in the study. The reasons for refusal were lack of energy or mental capacity or lack of time for being interviewed. None of the families that refused to participate differed from the participants regarding characteristics of the child’s medical condition or socio-demographic background.

Baseline physical and socio-demographic characteristics of mothers and children are shown in Table 1.

**Table 1:** **Socio-demographic and physical characteristics of the mothers given preterm birth and their children**

Mothers	n = 29
Age; mean (SD)	33.7 (4.3)
Education > 12 years; n (%)	26 (89.7)
Single parent; n (%)	0 (0)
Unemployed; n (%)	4 (13.8)
Previous psychological treatment; n (%)^a^	8 (27.6)
Chronic illness; n (%)^b^	2 (6.9)
Tot. no. of children; mean (SD)	1.7 (0.8)
Previous pregnancies; mean (SD)	1.1 (1.5)
Previous childbirths; mean (SD)	0.5 (0.7)
First time mothers; n (%)	18 (62.1)
IVF pregnancy; n (%)^c^	8 (27.6)
Bleeding in pregnancy; n (%)	19 (65.5)
Preeclampsia; n (%)	4 (14.3)
Pregnancy infection; n (%)	12 (41.4)
Cesarean emergency; n (%)	14 (48.3)
Cesarean planned; n (%)	3 (10.3)
Vaginal birth; n (%)	12 (41.4)
Breech birth; n (%)	3 (8.6)
**Children**	**n = 35**
Girl; n (%)	17 (48.6)
Boy; n (%)	18 (51.4)
Twin; n (%)	14 (40.0)
Gestational age (weeks);	
Median (range)	29 (24–32)
Mean (SD)	28.5 (2.6)
Birth weight (kg);	
Median (range)	1.2 (0.6-2.0)
Mean (SD)	1.2 (0.4)
Apgar score at 1 minute; median (SD)	6.3 (2.3)
Apgar score at 5 minutes; median (SD)	7.6 (2.0)
Apgar score at 10 minutes; median (SD)	8.3 (1.0)
Mechanical ventilation > 24 hours; n (%)	8 (22.9)
Oxygen supply > 28 days; n (%)	19 (54)
IVH grade 1 and 2; n (%)^d^	5 (14.3)
IVH grade 3 and 4; n (%)^d^	2 (5.7)
Surgery	4 (11.4)
Infection	7 (20.0)
Patent ductus arteriosis	6 (17.1)

^a^Everyone who had been in psychotherapy as a child or as an adult was registered.

^b^Diabetes and Crohn’s disease.

^c^In vitro fertilization/Assisted fertilization.

^d^Intraventricular hemorrhage following birth.

### Maternal mental health problems

The mothers in the study group reported significant mean scores in the clinical important range for psychological distress in all items on IES, GHQ and STAI (Table 2). Regarding the IES subscales, the Intrusion subscale showed the highest mean score. The proportion of Intrusion case score were 65.5%, while the proportion of Avoidance case score was 27.6% (Table 3).

**Table 2:** **Psychological distress, anxiety and trauma related stress reaction in mothers given preterm birth**

	Mean (standard error)
	n = 29
**Impact of event scale**	
Sum total score (0–75)	19.66 (2.00)
Sum Intrusion score (0–35)	14.00 (1.48)
Sum Avoidance score (0–40)	5.66 (0.81)
**General health questionnaire**	
Sum GHQ Likert score (0–90)	40.10 (2.90)
Sum GHQ case score (0–30)	12.76 (1.40)
State anxiety inventory*	
Sum total score (0–40)	21.45 (0.53)

*10 question version of STAI-X1.

**Table 3:** **Proportion of clinically important psychological distress, anxiety and trauma related stress**

	n = 29
Impact of event scale	
Case total ≥19	44.8%
Case intrusion, moderate (9–19)	31.0%
Case intrusion, severe ≥20	34.5%
Case avoidance, moderate (9–19)	27.6%
General health questionnaire	
Case total, ≥6	79.3%
State anxiety inventory*	
Case total, >20	59.0%

*10 question version of STAI-X1.

The clinical diagnosis assessment revealed that 28% of the mothers had depression, 17% had anxiety and 52% PTSR. Twenty-one percent of the mothers had more than one diagnosis.

### Associations between physical variables and maternal mental health problems

There were significant bivariate associations between several physical variables (planned Cesarean section, GA, birth weight, Apgar score, need of mechanical ventilation, patent ductus arteriosis (PDA), neonatal surgery), mother’s education, trait anxiety, parity, and previous psychological problems (Table 4). Increased maternal mental health problems were significantly associated with the mother’s education, trait anxiety, parity and the child’s GA, birth weight, and Apgar score. Other physical problems in pregnancy like planned Caesarean section and physical neonatal complications (mechanical ventilation, neonatal surgery, patent ductus arteriosis) were inversely associated with maternal mental health problems.

**Table 4:** **Significant correlations between socio-demographic, physical, and maternal mental health outcome variables**

	IES	GHQ^d^	STAI-X1
**Mother**			
Age			0.32′
Education (years)			0.51**
Previous psychological treatment		0.30′	
Parity			0.42*
Trait anxiety (STAI-X2)	0.32′	0.49**	
**Pregnancy/birth**			
Infection in pregnancy		−0.34*	−0.30′
Planned Caesarean	−0.36*		
Breech birth	0.31′		
**Child**			
Sex			−0.30′
GA			0.52**
Birth weight			0.39*
10 min Apgar score			0.38*
Mechanical ventilation			−0.43**
IVH^a^	0.30′		
Surgery^b^			−0.37*
Patent ductus arteriosis^c^			−0.42*

Pearson’s correlation coefficients (p < 0.10) are reported. (N = 35).

‘Correlation is significant at the 0.10 level (two-tailed).

*Correlation is significant at the 0 .05 level (two-tailed).

** Correlation is significant at the 0 .01 level (two-tailed).

^a^Intraventricular haemorrhage grade 1 or 2.

^b^Surgery after birth e.g. patent ductus arteriosis or hypospadias.

^c^Patent ductus arteriosis closed by medical treatment, surgery or by itself.

^d^The GHQ in this table refers to Likert sum scores.

At the 10% statistical significance level previous psychological treatment and intraventricular hemorrhage (IVF) grade 1 or 2 were associated with increased maternal mental health problems measured by the GHQ and the IES scale, respectively.

### Predictors of maternal mental health problems

The results of the forward multiple regression analyses are shown in Table 5. There was one significant predictor of the IES. “Planned Caesarean section” explained 10% of the variance in the IES (*p* < 0.05). Planned Caesarean was negative ( ) meaning that planned Caesarean section predict low IES scores. “Maternal trait anxiety” measured by STAI-X2 and “Other infection in pregnancy than preeclampsia” were significant predictors of GHQ Likert sum scores and explained 30% of the variance (*p* < 0.01, *p* < 0.05*)*. While “Maternal trait anxiety” predicted high GHQ Likert sum scores ( ), infection in pregnancy predicted low GHQ scores ( )*.* The significant predictors of STAI-X1 were the “Child’s Gestational Age (GA) at birth” and “Parity”, explaining 41% of the variance (*p* <0.001 and *p* < 0.01, respectively).

**Table 5:** **Forward entry multiple regression analyses predicting mental health outcomes for mothers after preterm birth**

Dependent variable	Independent variable	B	95% CI	Beta	p-value	R^2^_adj_
IES	Planned Caesarean section	−14.46	(−27.67, −1.25)	−0.36	0.033	0.10
GHQ	Maternal trait anxiety	1.97	(0.80, 3.14)	0.49	0.002	
	Infection in pregnancy	- 5.14	(−9.79, −0.49)	−0.32	0.031	0.30
STAI-X1	GA (weeks in pregnancy)	0.57	(0.28, 0.86)	0.52	0.000	
	Parity	0.42	(0.63, 2.79)	0.42	0.003	0.41

Unstandardized *(B)* and standardized *(Beta)* regression coefficients. (N = 35).

A further forward multiple regression analysis was performed of the IES subscales. The results yielded “Planned Caesarean section” as a predictor of low IES Intrusion sum scores (*p* < 0.05), and “Vaginal delivery” was found to be a predictor of high IES Avoidance sum scores (*p* < 0.05). The two predictors explained 8% (R^2^adjusted = .08) and 10% (R^2^adjusted = .10), respectively, of the variance.

## Discussion

The present study revealed that mothers who delivered preterm reported levels of psychological distress in the clinically important significant range two weeks after delivery.

In this study we detected that 52% of the mothers showed traumarelated symptoms and less than one third of the mothers showed symptoms of depression two weeks after the preterm delivery. The predictors for maternal mental health problems following preterm birth were related to the pregnancy, the preterm delivery, the child’s GA, the maternal trait anxiety, and parity.

Epidemiologically high levels of mental health problems has been revealed [31, 32]. The prevalence of mental health problems in Norway is reported to be in accordance with prevalence in other European countries; about 30% in a 12-month period and about 50% in a lifetime span [32–34]. The high prevalence of maternal stress and anxiety following preterm delivery in our study corresponds with previous studies [5–8]. Our results are considerably higher than expected when we compare them with other pure samples of patients with physical illness only [26]. Whether psychological distress should be seen as a normal reaction to preterm birth or not is still an open question. Compared with another Norwegian study of mothers giving birth at term for instance we found a prevalence of clinically important maternal psychological distress of 79%, while they reported 37% [35]. In addition, the same study detected that 9% of the term mothers reported severe intrusive stress symptoms in IES. In comparison 34.5% of the preterm mothers in our study reported severe intrusive stress symptoms in IES.

The clinical diagnostic assessment of the preterm mothers showed that posttraumatic stress reactions (PTSR) represented the most common reactions. The prevalence of PTSR, depression, and anxiety was 52%, 28%, and 17%, respectively. Our results are comparable to a study that reported 49% significant trauma reactions among mothers one year after delivery [20], but differ from another study which revealed a relation between high levels of maternal posttraumatic stress symptoms (>33%) and high levels of depression (>53%) [36]. Interestingly we revealed a high prevalence of severe intrusion stress symptoms measured by IES. Our result is comparable to another study that reported higher mean scores in the IES intrusion subscale in a preterm sample at discharge from the hospital [20]. Our study showed that preterm delivery may represent a significant trauma experience for the mother and that the prevalence of depression and anxiety was low compared to post-traumatic stress responses.

Planned Caesarean section was found to predict both low IES sum scores in the forward multiple regression analysis. In the study group, most of the Caesareans were emergency, but for a few hospitalized mothers with different physical issues like infections or growth retardation in fetus, the Caesarean section was planned some time in advance, and they had at least about a week to prepare themselves for the procedure. The results of the IES analyses may indicate that time to prepare oneself of a preterm birth as a planned Caesarean could reduce the traumatic stress response.

The child’s gestational age (GA) at birth and maternal parity were the predictors of maternal stress and anxiety state levels (STAI-X1). It is noteworthy that numbers of previous childbirths are related to high anxiety state sum scores. The association between high GA and high levels of state stress and anxiety corresponds with findings in a study of term birth with ultrasound detections [37]. A Norwegian study on maternal psychological responses after ultrasound scan at 18 weeks of GA, with or without detection of fetal anomalies, also reported that advanced GA at diagnosis of fetal anomalies was one of the strongest predictors of psychological distress [38].

There may be several variables that could explain why advanced GA predict high levels of psychological distress. The preparation for the motherhood is close in time and first time mothers may worry about their limited experiences with parenthood. In addition, our results may be hypothesized to be a result of growing relationship to the fetus during pregnancy [39, 40]. The “fear of losing the baby” evoked by a preterm birth and “growing relationship” to the baby are probably closely related. Several studies have focused on preterm deliveries and the “fear of losing the baby”, and on the relationship between the “intensity of grief” and increasing GA, when mothers have experienced pregnancy termination or pregnancy loss [41–43].

A recent study including an attachment research instrument (Adult Attachment Projective) revealed that 67% of mothers that had experienced a preterm birth showed unresolved posttraumatic issues regarding the “anxiety of losing the baby” 6 years after the preterm birth [42, 44]. In attachment theory, a “threat” is understood as a crucial element in the onset of human behavioral strategies [45] and psychological distress might be the result when the strategies do not work or do not eliminate the “threat”. “fear of losing the baby” is likely to be experienced as a “threat” in a psychological sense. Attachment theory might well represent a theoretical framework that could broaden our understanding of the high levels of the maternal mental health responses after a preterm birth in our study.

Personal vulnerability factors such as previous mental health problems and trait anxiety were associated with general psychological distress in the GHQ Likert sum scores, as well as being significant predictors of general psychological distress measured by GHQ Likert sum scores. Our finding corresponds with another study that explored the prevalence of post-traumatic stress symptoms following childbirth [46]. Thus, the maternal previous mental health history seems to be important to include in the medical charts of mothers that deliver at preterm when assessing the mother’s need of psychological support after birth.

Our study revealed an inverse association between pregnancy complications and psychological distress, anxiety and trauma related stress. Pregnancy complications do not measure the mother’s “fear of losing the baby” explicitly, but may be an indicator of this phenomenon. Indeed the pregnancy complication variables “planned Caesarean section”, and “infection in pregnancy” turned out to be the predictors of low maternal psychological distress and traumarelated stress scores in the IES and the GHQ. A planned Caesarean implies more control in labor than an emergency Caesarean or a spontaneous vaginal delivery. Previous studies that have explored the association between maternal distress and physical complications in pregnancy and in infants in the perinatal or postnatal period have reported results that run counter to those of our study [41, 47–52].

Some of the results in our study have been difficult to explain but should be mentioned for future research purposes. For instance, we found an significant association in our correlation analysis between lower maternal mental health problems in STAI-X1 and two medical conditions the child was treated for after birth; the common condition patent ductus arteriosis (PDA) and the more severe hypospadias condition.

Medical conditions that require treatment and surgery may easily be assumed as distressing for the parents. Why these conditions lower the maternal anxiety in the STAI-X1 scale is not obvious, but may be explained by the extended support to the parents that the medical staff are likely to give in such an occasion. Our study also detected an association at the 10% significant level between intraventricular hemorrhage (IVF) grade 1 or 2 and high levels of maternal traumarelated problems measured by IES (Table 4). IVF grade 1 or 2 is less severe than grade 3 or 4 which may be reflected in the level of support to the parents from the medical staff. The parents on the other hand could be distressed by the IVF grade 1 or 2 condition and be worried that the IVF incidence will have an negative influence on their baby’s development.

### Strength/limitations

The preterm participants came from well-defined geographic areas and were included consecutively in the study, thus minimizing selection bias. The response rate is high. The psychometric instruments (IES, GHQ, STAI) used in our study are all well-validated. We have assessed several important aspects of mental health problems, like psychological distress, anxiety, and trauma-related stress and also assessed tentative clinical diagnosis for the prevalence of mental health problems.

The present study describes a small group of mothers giving preterm birth with higher educational attainments, older age and higher socioeconomic status than would be found in a typical population of mothers who deliver preterm in Norway. Thus, one should be cautious about generalizing from this study. The homogeneity of the study group in terms of socio-demographic background and distress related to it is clearly a limitation in this study. On the other hand our results were controlled for highrisk socio-economic background variables. A more detailed assessment of maternal trauma related experiences prior to the pregnancy would of course have strengthened our study. However, data was collected of the study group’s mental history and about their pregnancy experiences.

A tentative clinical diagnosis based on information from the standardized self reports was assessed by a psychiatrist. A diagnostic semistructured interview, however, would have been preferable in addition to information from psychometric self-report questionnaires to make a correct clinical diagnosis.

## Conclusions

Our study revealed a substantial level of psychological distress in mothers who deliver preterm. Posttraumatic stress was the most common psychological reaction to preterm delivery in this study. Significant physical predictors that were discovered should be further investigated. Our findings imply that mothers’ psychological reactions to preterm birth need to be taken into account at maternity wards. The need for intervention and psychotherapy should be considered at an early stage and while the infant still is in the NICU. It is, however, necessary to investigate these results closer in a prospective and longitudinal study as these psychological patterns might change over time.
